# A Case Report of the Coexistence of Gastric Cancer With Polycystic Kidney and Liver Disease: Unveiling the Complexity

**DOI:** 10.7759/cureus.53574

**Published:** 2024-02-04

**Authors:** Amit Girme, Vernika Gupta

**Affiliations:** 1 General Surgery, Dr. D. Y. Patil Medical College, Hospital & Research Centre, Pune, IND

**Keywords:** early detection and prevention, esophagogastroduodenoscopy, ultrasonography, gastric cancer, polycystic kidney and liver disease

## Abstract

Polycystic kidney disease (PKD) is a genetic disorder that comprises multiple cystic lesions in the kidneys. The association of PKD with gastric cancer has been studied. We present a rare presentation of stomach cancer with polycystic liver and kidney disease. A male patient in his 40s presented with epigastric pain, nausea, retrosternal burning, and occasional episodes of vomiting. Esophagogastroduodenoscopy revealed ulceroproliferative growth in the prepyloric region. Biopsies revealed moderately differentiated adenocarcinoma which was confirmed by contrast-enhanced computed tomography of the abdomen and pelvis. This showed a chance finding of polycystic kidney and liver disease. After confirmation with a positron emission tomography scan, the patient was diagnosed with gastric carcinoma (cT3N1M0, Stage IIB) with polycystic kidney and liver disease. We provide a case of early-stage stomach cancer in a patient with PKD. More extensive research is needed for a better understanding of this association between polycystic kidney and liver disease and gastric cancer development, to achieve earlier diagnosis.

## Introduction

In the intricate landscape of medical pathology, instances of rare and concurrent conditions often pose unique challenges for clinicians and researchers alike. This case report sheds light on an extraordinary intersection of diseases, documenting the presence of gastric cancer alongside polycystic kidney and liver disease in a single patient. This remarkable convergence of pathologies not only underscores the importance of comprehensive diagnostic approaches but also prompts a deeper exploration into potential shared genetic, molecular, or environmental factors contributing to their coexistence.

Gastric cancer, a formidable malignancy with a diverse spectrum of presentations, stands as one of the leading causes of cancer-related morbidity and mortality worldwide [1]. Concurrently, polycystic kidney and liver disease, a genetic disorder characterized by the formation of cysts within these vital organs [2], introduces another layer of complexity to the clinical scenario. While each condition individually demands meticulous attention and tailored management, the juxtaposition of gastric cancer with polycystic kidney and liver disease raises intriguing questions about the intricate interplay of these disparate pathologies. To our knowledge, only eight cases have been reported so far which discuss the correlation and pathophysiology of gastric cancer's association with polycystic disease of the kidneys [3-9].

This case report aims to present a comprehensive analysis of the patient's clinical history, diagnostic journey, and subsequent management. By delving into the molecular and genetic aspects, as well as exploring potential connections between these conditions, we endeavor to contribute to the evolving understanding of disease associations and pave the way for more informed and targeted diagnostic and therapeutic strategies. As we navigate through the nuances of this rare clinical entity, we invite the medical community to join us in the pursuit of knowledge, ultimately enhancing our ability to diagnose, treat, and perhaps prevent such complex comorbidities in the future.

## Case presentation

A male patient in his 40s presented to the General Surgery OPD of Dr. D.Y. Patil Hospital, Pune with epigastric pain, nausea, retrosternal burning, and occasional episodes of vomiting. He was advised esophagogastroduodenoscopy (EGD).

**Figure 1:**
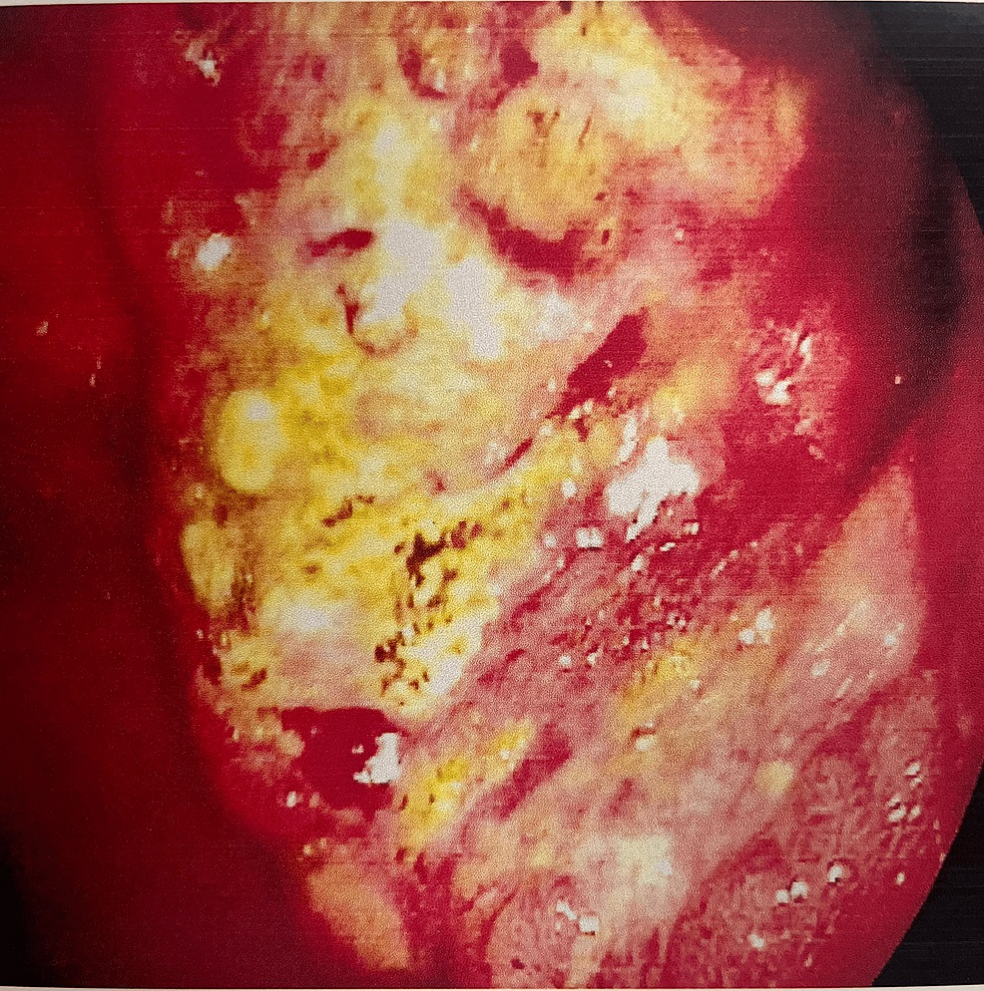
Esophagogastroduodenoscopy image of the growth present in the prepyloric region.

EGD revealed an ulceroproliferative growth in the prepyloric region extending into the pylorus with necrotic slough present at its base as depicted in Figure 1. The pyloric lumen seemed to be narrow, and the scope couldn’t be negotiated further. A rapid urease test showed negative results.

Multiple biopsies were taken from the growth and sent for histopathological examination which revealed moderately differentiated adenocarcinoma.

**Figure 2:**
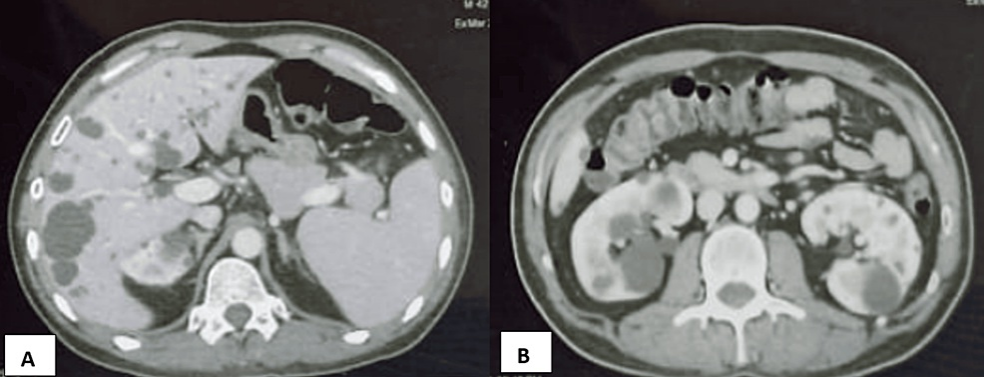
CECT images showing polycystic (A) liver and (B) kidney disease. CECT: Contrast-enhanced computed tomography

In the following days, the patient was subjected to further evaluation. He had an abnormally high carcinoembryonic antigen value (52.37). The contrast-enhanced computed tomography (CECT) scan showed an asymmetrical circumferential wall thickening involving the antropyloric region of the stomach with a maximum thickness of 16 mm and an approximate length of involvement measuring 43 mm. The lesion was causing luminal narrowing with mild dilatation of the proximal stomach. The fat plane between the lesion and medial margin of segment V of the liver was lost, but fat planes between the pancreas and gall bladder were maintained. The lesion was reaching up to and closely abutting the first part of the duodenum which was partially distended with maximum wall thickness measuring 5 mm along the superior aspect. CT incidentally revealed multiple renal and liver cysts s/o polycystic kidney and polycystic liver disease as depicted in Figure 2.

**Figure 3:**
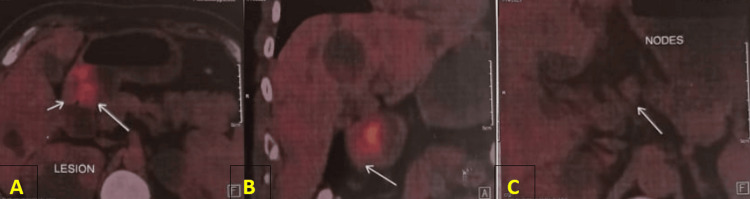
8-FDG PET scan images showing (A and B) asymmetric thickening in the antropyloric region with (C) weakly metabolic regional nodes. 8-FDG PET: 8-Fluorodeoxyglucose positron emission tomography

8-Fluorodeoxyglucose (FDG) PET done for metastatic workup revealed asymmetric thickening in the antropyloric region with weakly metabolic regional nodes (peripyloric and gastrohepatic) without any distant organ involvement as depicted in Figure 3.

Based on these findings, the patient underwent staging laparoscopy which revealed no peritoneal metastasis, no locally advanced lesion, and no serosal involvement. A peritoneal wash was given and the fluid aspirated was sent for malignant cytology, which was later reported as free of malignancy.

Diagnosis

Based on the above evaluation, the patient was diagnosed with gastric carcinoma (cT3N1M0, Stage IIB) with polycystic liver and kidney disease.

Outcome and follow-up

The patient took four cycles of neoadjuvant chemotherapy. He underwent a D2 gastrectomy followed by another four cycles of adjuvant chemotherapy. The patient was followed up after surgery and after every chemo cycle. He does not have any symptoms at present and is living a healthy lifestyle.

## Discussion

We outline a rare case of stomach cancer associated with polycystic liver and kidney disease. Informed and written consent was taken from the patient before enrolling him in this case study.

Polycystic disease of the kidney is classified as an autosomal dominant form (more common, age of presentation: 30-50 years) and an autosomal recessive form (less common). Autosomal dominant polycystic kidney disease (ADPKD) can be associated with cysts in the pancreas, liver, arachnoid, and seminal vesicles [10]. Cyst formation in ADPKD is caused by mutations in the PKD-1 (16p13.3) and PKD-2 (4q22.1) genes that lower intracellular PC-1 or PC-2 protein levels. Both genes, respectively, are mutated in 85% and 15% of ADPKD patients [11].

Yasuda et al. conducted a population-based cohort which reported that PKD patients had a higher chance of developing gastric cancer [8]. In such patients, the cancer diagnosis and prognosis are related to the proliferation of cells and their differentiation, migration, polarity, and apoptosis [12]. PKD-1 encodes PC-1, an enormous glycoprotein spanning the cell membrane [13].

PC-1 spreads all through the body. It is exceedingly communicated within the liver, kidneys, guts, pancreas, heart, and brain [14]. It, moreover, plays a role in modulating cell-to-cell and cell-to-matrix interactions, proliferation of cells, apoptosis, and transport of cations. It interacts with intestinal E-cadherin. Mutations in PKD-1 lead to decreased function of PC-1, thus, leading to poor cell adhesion and polarity, ultimately causing unconstrained proliferation of cells [11]. PC-1 has been implicated in various kinds of solid carcinoma as cell growth, invasive growth, and metastatic growth suppressor [15,16].

Defects in many signaling pathways, including the mTOR signaling pathway, are believed to be associated with dysfunction of PC-1 or PC-2, which mostly increase cyst development in ADPKD patients, whereas mTOR inhibitors cause renal cyst reduction in said individuals. In particular, mTOR signaling is upregulated in a lot of malignancies leading to increased chances of metastasis and resistance to pharmaceuticals. As a result, mTOR has been considered a new target for cancer therapy. This mTOR signaling pathway is recognized to be dynamic in gastric cancer patients. Its activation has been linked to tumor development and a bad prognosis [17].

These discoveries detailed by past considerations propose at least a halfway cover between gastric carcinogenesis, metastasis, and ADPKD development concerning variations at the molecular level. Table 1 depicts a comprehensive summary of the past studies with individual management and the outcome of each case.

**Table 1:** Summary of case reports presented with gastric cancer associated with polycystic kidney disease documented globally. PKD: Polycystic kidney disease

No.	Authors	Year	Age/Sex	Family history of PKD	Stage of gastric cancer	Outcome	References
1	Aziz	1998	40yr/M	Sister	Unknown	Dead	[3]
2	Aziz	1998	50yr/F	Brother	Unknown	Dead	[3]
3	Tsukayama	2002	68yr/M	Mother, brother	Stage IA	Alive	[4]
4	Yamanaka	2010	50yr/M	Unknown	Stage IV	Alive	[5]
5	Mimatsu	2011	65yr/M	Sister	Stage II	Alive	[6]
6	Kaya	2012	44yr/M	Three brothers	Unknown	Dead	[7]
			48yr/M		Unknown	Dead	
7	Yasuda	2020	60yr/M	Mother, brother	Stage IVB	Dead	[8]
8	Coco	2023	70yr/M	None	Stage IV	Alive	[9]
9	Our case	2023	42yr/M	Unknown	Stage IIB	Alive	-

Among all the case reports, the procedure of choice was D2 gastrectomy, when operated. No significant guidelines were found for the type of gastrectomy to be undergone by the patient. No study mentions the additional benefit of putting the patient through a total gastrectomy. Whether such patients should be taken up for prophylactic gastrectomy remains a point of controversy, and further research needs to be carried out in this regard.

## Conclusions

We provide a case of early-stage stomach cancer in a patient with PKD. We explored the likely pathomechanism of this illness because a statistical link between PKD and stomach cancer has already been described. Aberrant mTOR or defective PC-1 expressions are detected in ADPKD patients. Based on prior study findings, we hypothesized that these molecular abnormalities might have led to the development of stomach cancer in this case. More extensive research is required to acquire a better understanding of the ramifications of this association between polycystic kidney and liver disease with gastric cancer development and progression, to achieve earlier diagnosis and devise a plan of action for such patients.

Regular screening is required in patients with ADPKD. Patients detected with ADPKD can undergo early diagnosis of gastric cancer. Such patients can be subjected to prompt intervention. This will contribute to a better prognosis and an increased rate of survival of such patients. Relatives of such patients should be evaluated for similar occurrences due to genetic predisposition of the above-mentioned condition.
